# Friend or Foe: Prognostic and Immunotherapy Roles of BTLA in Colorectal Cancer

**DOI:** 10.3389/fmolb.2020.00148

**Published:** 2020-07-21

**Authors:** Jingjing Song, Lihui Wu

**Affiliations:** ^1^Department of Children’s Health Care, The Second Affiliated Hospital & Yuying Children’s Hospital, Wenzhou Medical University, Wenzhou, China; ^2^Department of Clinical Medicine, Hangzhou Medical College, Hangzhou, China

**Keywords:** BTLA, tumor-infiltration, prognosis, colorectal cancer, immunotherapy

## Abstract

**Background:**

B and T lymphocyte attenuator (BTLA) is a co-signaling protein belonging to the CD28 immunoglobulin superfamily. However, the role of BTLA in prognosis and immunotherapy of colorectal cancer (CRC) remains unclear.

**Methods:**

We evaluated the expression of BTLA via the Oncomine and the cancer genome atlas (TCGA) database. We research the outcome among different BTLA expression patients by Kaplan–Meier curve. We used the Chi-Squared test and Cox regression analysis to identify potential risk factors. Besides, the correlations between BTLA and cancer immune infiltration were investigated via CIBERSORT.

**Results:**

Various cohorts showed that BTLA expression was lower in CRC compared to corresponding normal tissue. Moreover, low BTLA expression was correlated with poor overall survival in TCGA cohorts and Gene Expression Omnibus cohorts (GSE29623 and GSE17536). Low BTLA expression was associated with less lymph node metastasis (*p* = 0.0123). In the Cox proportional hazards model, BTLA was identified as a favorable prognostic factor. Naive B cells, memory B cells, CD8 T cells, CD4 memory resting T cells, follicular helper T (Tfh) cells, monocytes, resting natural killing (NK) cells, M0 macrophages, M1 macrophages, resting mast cells, and activated mast cells were affected by BTLA expression (all *p* < 0.01). Correlated immune markers and functional enrichment analysis revealed BTLA functioned in the T cell receptor signaling pathway, B cell receptor signaling pathway, and NK cell-mediated cytotoxicity pathway.

**Conclusion:**

These analyses suggest BTLA is a potential factor for extended survival and closely related to CD8 T cells, Tfh cells, B cells, and NK cells in CRC. We summarize these results that BTLA can be used as a prognostic biomarker and might contribute to developing novel CRC immunological treatment strategies.

## Introduction

Colorectal cancer (CRC) was estimated to be the third leading component cause of cancer-related mortality in the world (Siegel et al., 2020). Up to now, surgical resection has been the most effective method for CRC. However, approximately 40% of CRC patients who were cured after the surgery will recur within five years (Augestad et al., 2017). Thus, other effective treatments are in urgent to improve the prognosis of CRC patients.

Immunotherapy has been postulated to be a more precise approach with high future potential, especially those with deficient mismatch repair (MMR) or high microsatellite instability (MSI-H). Major immunotherapy trials in metastatic CRC have been focused on selective anti- programmd death 1 (PD-1), anti-PD-L1, and anti-Cytotoxic T lymphocyte antigen 4 (CTLA-4) monoclonal antibodies. Immune checkpoint inhibitors are sure to have therapeutic effects supporting by plenty of clinical evidence, but the vast majority of patients with MMR or microsatellite stable (MSS) do not benefit from immunotherapy (Le et al., 2015; Bendell et al., 2016; Overman et al., 2018). Consequently, new effective immunotherapy targets, which can make the tumor more sensitive to immune checkpoint inhibitors and lend extensive immune infiltration, are in urgent need.

B and T lymphocyte attenuator (BTLA) is a co-signaling protein expressed by T cells, B cells, natural killer (NK) cells, and antigen-presenting cells. Herpes virus entry mediator (HVEM), a receptor of tumor necrosis factor family, is the known ligand for BTLA in mice and humans. Ligation of BTLA by HVEM has been shown to recruit SHP-1 and SHP-2 protein tyrosine phosphatases, resulting in suppression of T-cell receptor (TCR) activation (Watanabe et al., 2003; Murphy et al., 2006). B and T lymphocyte attenuator, CTLA-4, CD28, inducible costimulatory molecule (ICOS), and PD-1 are all belonging to the CD28 immunoglobulin superfamily (Ishida et al., 1992; Hutloff et al., 1999; Freeman et al., 2000; Watanabe et al., 2003). According to the previous research, combining PD-1 antibodies and BTLA antibodies might be of treatment effect in conditions in which tumor masses are non-homogeneous in terms of PD-1 and BTLA ligand expression (Celis-Gutierrez et al., 2019). Besides, BTLA can regulate γδT cell homeostasis, cytotoxic T-lymphocyte activity, and inflammatory cytokines production to suppress the inflammatory responses (del Rio et al., 2011; Bekiaris et al., 2013). In conclusion, BTLA exerts bidirectional regulatory effects: one is a kind of inhibitor on T lymphocytes with similarities to the well-known CTLA-4 and PD-1, the other is a positive stimulator like those on ICOS and CD28 proteins.

No previous study has reported the mechanism of BTLA-mediated immune infiltration of CRC. Therefore, we used online databases to explore the expression of BTLA firstly. Then the cancer genome atlas (TCGA) database, which contains CRC samples providing a large number of clinical and transcriptome data, was used for further research. The unambiguous correlation of BTLA with the prognosis of CRC was concluded. Cell-type identification by estimating relative subsets of RNA transcripts (CIBERSORT) was used to obtain the fractions of tumor-infiltrating immune cells (TIICs) in the tumor microenvironment (TME) in our analysis (Chen et al., 2018). We found high expression of BTLA was closely related to a higher proportion of a lot of TIICs. Moreover, we analyzed the correlation between immune maker genes and BTLA and combined the gene ontology (GO) and Kyoto Encyclopedia of Genes and Genomes (KEGG) functional process. Our findings disclose that BTLA may be a possible target for CRC immunotherapy in our present study.

## Materials and Methods

### Expression Analysis

The transcriptome expression profiles and relevant clinical information of CRC were all downloaded from the TCGA database (Weinstein et al., 2013). The expression data was HiSeq-RSEM type, including 610 CRC tissues and 51 adjacent non-tumorous tissue samples. Tumor Immune Estimation Resource (TIMER) analyzes TIICs in over 10,000 RNA-seq TCGA database samples across 32 cancer types (Li et al., 2017). In this research, the transcriptional level of BTLA in several tumors was analyzed and compared via TIMER databases. Oncomine database is one of the largest cancer microarray databases and web-based data-mining comprehensive platform discovering from genome-wide expression analyses (Rhodes et al., 2004). In this research, the expression level of BTLA between tumor tissues and normal tissues were analyzed and compared via Oncomine databases. Cut-off of p and fold change were set as the following: *p* = 0.05, fold change = 1.5, gene rank = all. DNA and mRNA subtypes data types were both included in this analysis.

### Survival Analyses

An integrated TCGA clinical data resource was downloaded to perform high-quality survival analytics among CRC samples (Liu et al., 2018). Gene Expression Omnibus (GEO) datasets were downloaded to validate the accuracy of the TCGA cohort. Raw microarray expression public data GSE29623 and GSE17536 of CRC were selected. The probe 236226_s_at was used in this research matching for BTLA. Kaplan-Meier survival curves and the log-rank test were used to evaluate the significance of survival time differences by “survival” and “survminer” R package. The “maxstat” R package determined the best cutoff. Univariate and multivariate Cox regression analyses were carried out to identify overall survival (OS)-related clinical characteristics and potential interactions among the factors. Cox regression results were kept in two significant digits.

### Evaluation of Immune Cell Infiltration

All malignancy tissues from TCGA were retained to perform CIBERSORT analysis to study the influences on immune cell infiltration by BTLA expression. The differential infiltrations of the 22 immune cell types in the CRC samples were evaluated by CIBERSORT R script (version 1.03). One thousand permutations were set for the default signature matrix using the algorithm. The criterion *p* < 0.05 was set to select the cases in the subsequent analysis. The correlation between the BTLA level and the profusion of immune cells was analyzed by the “vioplot” R package. Associations among different TIICs were evaluated by the “corrplot” R package. Furthermore, correlations between BTLA expression and immune gene marker sets were calculated via the “Correlation” function by Graphpad Prism 8.0.2.

### Functional Enrichment Analysis

The correlation analysis was assessed with the Spearman method by function “cor.test” in R. The genes that had highly ranked positive or negative correlation coefficients with BTLA were selected. “enrichGo” and “enrichKEGG” functions in “clusterprofiler” R package was used to perform GO and KEGG analysis, respectively. “ggplot2” and “pheatmap” R package was used to visualization.

### Statistical Analysis

All analyses were conducted by R software (version 3.5.3). The two-tailed paired *t*-test was used for data of expression of BTLA. The correlations between BTLA expression and clinical characteristics were analyzed using the Chi-Squared test. Pearson’s correlation test was used to test correlations between gene marker and BTLA expression. All statistical analyses were two-sided, and *p* < 0.05 were considered as statistically significant. The original data were extracted from TCGA and GEO, which were openly available and free of access barriers, so there was no requirement for ethics committee approval.

## Results

### The Expression Level of BTLA

**FIGURE 1:**
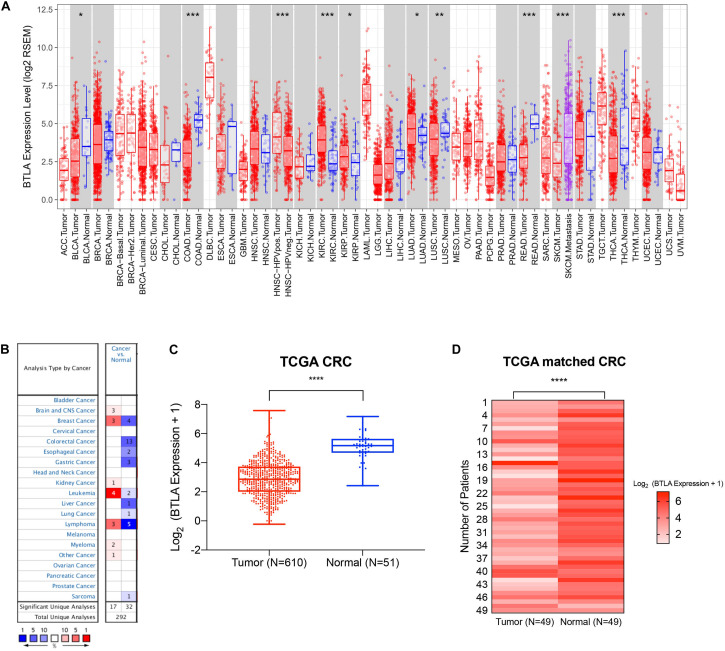
The expression of BTLA in colorectal cancer and other cancer types. **(A)** BTLA mRNA levels in different cancer types by TIMER database. *p < 0.05, **p < 0.01, ***p < 0.001. **(B)** BTLA in different cancer data sets increased or decreased compared with normal tissues detected by the Oncomine database. **(C)** BTLA expression levels in whole tumorous tissues and normal tissues in TCGA cohorts. ****p < 0.0001. **(D)** BTLA expression levels in tumor tissues and paired normal adjacent tissues in TCGA cohorts.****p < 0.0001.

We first screened and compared the expression of BTLA in different cancer types. The TIMER database analysis revealed that BTLA mRNA expression was lower in bladder cancer (BLCA), colon adenocarcinoma (COAD), lung squamous cell carcinoma (LUSC), rectum adenocarcinoma (READ), and thyroid carcinoma (THCA) compared with adjacent normal tissues. Higher expression was observed in kidney renal clear cell carcinoma (KIRC), kidney renal papillary cell carcinoma (KIRP), lung adenocarcinoma (LUAD) compared with adjacent normal tissues (Figure 1A). In addition, the Oncomine database showed BTLA decreased in 13 CRC data sets compared to the normal tissues (Figure 1B), the trends were in line with previous TIMER database results. To further evaluate the BTLA expression of CRC, we analyzed BTLA expression using TCGA RNA sequencing data. BTLA mRNA levels were significantly lower in cancerous tissues (Figure 1C), as well as in paired tumor and normal samples (Figure 1D).

### Correlation of BTLA Expression With the Clinicopathological Characteristics of Colorectal Cancer

To further explore the BTLA expression in CRC, we use the TCGA cohort to analyze the underlying mechanism and correlate it with certain clinical aspects. CRC samples with qualified clinical information were analyzed using the Chi-Squared test, hence revealing that higher expression of BTLA significantly correlated with the lower grade of lymph node metastasis (Table 1).

**TABLE 1:** Correlation of BTLA mRNA expression and different clinicopathological factors by Chi-Squared Test.

	BTLA expression		*p*
	Low (*N* = 303)	High (*N* = 304)	
Age (years)			0.16
<60	79	95	
≥60	224	209	
Gender			0.54
Male	165	158	
Female	138	146	
T classification			0.52
T1 + Tis	10	11	
T2	45	58	
T3	211	202	
T4	37	32	
N classification			0.25
N0	164	183	
N1	78	67	
N2	61	51	
M classification			0.012
M0	217	233	
M1	53	31	
Stage (AJCC)			0.075
I	42	61	
II	114	113	
III	90	86	
IV	52	35	

### BTLA mRNA Levels Predict Prognosis in Colorectal Cancer

**FIGURE 2:**
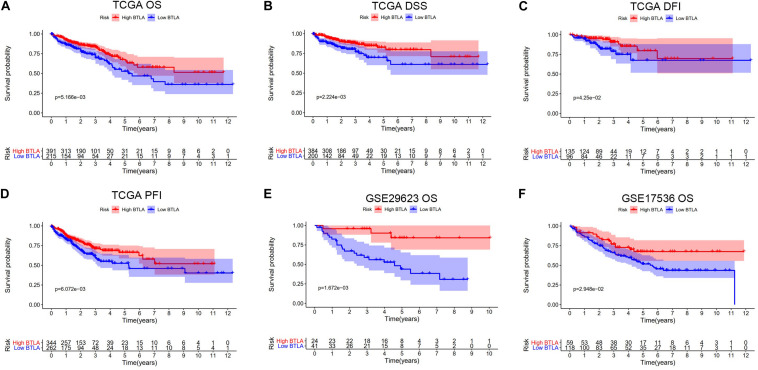
Kaplan-Meier survival curves comparing the different expression of BTLA groups in colorectal cancer in TCGA cohorts and GEO cohorts. **(A–D)** Survival curves of OS, DSS, DFI, PFI in TCGA colorectal cancer cohorts. **(E,F)** Low BTLA expression was correlated with poor OS in the GEO cohorts. OS, overall survival; DSS, disease-specific survival; DFI, disease-free interval; PFI, progression-free interval.

We compared OS, disease-specific survival (DSS), disease-free interval (DFI), and progression-free interval (PFI) between high BTLA expression group and low BTLA expression group in TCGA cohorts. As shown in Kaplan-Meier survival curves, reduced expression of BTLA was significantly correlated with the poor OS, DSS, DFI, and PFI (Figures 2A–D). Besides, we used different CRC cohorts, including GSE29623 and GSE17536, also showed that lower BTLA expression markedly correlated with a poor OS (Figures 2E,F). After deleting incomplete clinical samples, 514 TCGA tumor tissues with clinical details were finally devoted to Cox regression analysis. In the univariate Cox regression model, the TNM stage, AJCC stage, and age were significant prognostic factors relevant to OS, *p* < 0.05 (Table 2). Importantly, lower BTLA expression obviously correlated with poor OS (HR = 0.46, *p* < 0.001). Then, multivariable Cox regression analyses were performed to confirm the significant prognostic factors. After adjusting the known risk factors, the Cox proportional hazards model of OS was built, respectively. Similarly, the up-regulated BTLA expression, less than 60 years old, along with the lower T, N stage were independent prognostic factors of favorable prognosis (Table 2 and Supplementary Figure S1).

**TABLE 2:** Univariate Cox analysis and Multivariate Cox analysis of BTLA expression and other clinicopathological factors.

Variable	Univariate Cox Regression				Multivariate Cox Regression			
	β	HR (95% CI)	*Z* score	*p*	β	HR (95% CI)	*Z* score	*p*
Age: ≥60 vs <60	0.52	1.7 (1.0–2.8)	4.0	0.045**	0.70	2.0 (1.2–3.4)	2.6	0.0089**
Gender: Female vs male	0.14	1.1 (0.77–1.7)	0.45	0.50				
Disease stage: high vs low	0.83	2.3 (1.8–2.9)	51	1.10E-12***	0.064	1.1 (0.56–2.0)	0.19	0.85
T stage: high vs low	1.1	3 (2.0–4.4)	28	1.40E-07***	0.75	2.1 (1.3–3.4)	3.2	0.0013**
N stage: high vs low	0.76	2.1 (1.7–2.7)	42	1.10E-10***	0.51	1.7 (1.1–2.5)	2.5	0.011*
M stage: high vs low	1.5	4.3 (2.8–6.6)	47	8.40E-12***	0.75	2.1 (0.86–5.2)	1.6	0.10
BTLA expression: high vs low	−0.77	0.46 (0.31–0.70)	13	0.00027***	−0.62	0.54 (0.35–0.82)	−2.9	0.0037**

*p < 0.05; **p < 0.01; ***p < 0.001.

### BTLA mRNA Levels Are Associated With Tumor-Infiltrating Lymphocytes

**FIGURE 3:**
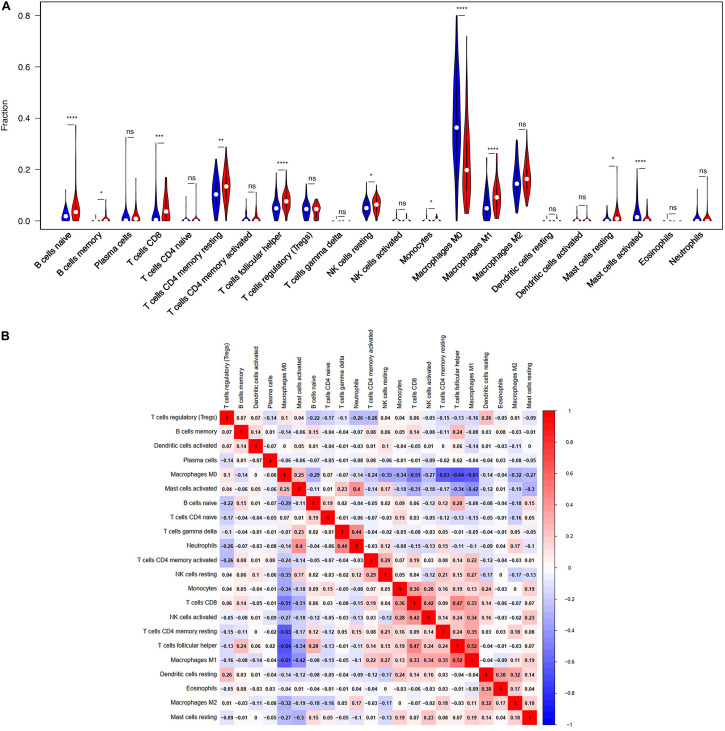
BTLA-related immune infiltration alteration. **(A)** The group with high expression of BTLA is red, and the group with low expression of BTLA is blue. Naive B cells, memory B cells, CD8 T cells, CD4 memory resting T cells, follicular helper T cells, resting NK cells, M1 macrophages and resting mast cells share a higher proportion in high expression group compared with low expression group. On the contrary, the proportion of monocytes, M0 macrophages, activated mast cells are apparently lower. **(B)** The proportions of different immune infiltration cells subpopulations were weakly to highly correlated. ns, *p* > 0.05; *p < 0.05; **p < 0.01; ***p < 0.001; ****p < 0.0001.

TIICs are miscellaneous in tumorous tissue and adjacent tumorous tissue and may have totally different functions and effects on survival, which can vary according to cancer types (Fridman et al., 2012). For this reason, we wanted to figure out whether the expression of BTLA was related to TIICs levels in CRC. CIBERSORT, a versatile computational method for estimating the abundances of member cell types, was used to quantify the fraction of TIICs in high and low BTLA expression groups. Validation of CIBERSORT for determining TIICs composition and their correlation with the prognosis has been confirmed in breast, lung, and renal cancers successfully (Ali et al., 2016; Chen H. et al., 2019; Zhang S. et al., 2019). The output p of global deconvolution was evaluated for each sample, and only *p* < 0.05 samples were used for the following research. Finally, 148 samples met the screening criterion and were respectively divided into high BTLA expression group and low BTLA expression group. The proportions of 22 subtypes of TIICs of CIBERSORT were clearly exhibited in Figure 3. Naive B cells, memory B cells, CD4 memory resting T cells, CD8 T cells, follicular helper T (Tfh) cells, monocytes, resting NK cells, M0 macrophages, M1 macrophages, resting mast cells, and activated mast cells were affected by BTLA expression (all *p* < 0.05). Among them, most cells shared a higher percentage in the high BTLA expression group compared with the low expression group. Conversely, the proportion of monocytes (*p* = 0.024), M0 macrophages (p < 0.0001), and activated mast cells (*p* < 0.0001) were infiltrated significantly less in high BTLA expression group compared with the other group (Figure 3A). As shown in Figure 3B correlation heatmap, the proportions of different TIICs subpopulations had weak to high correlation in tumorous tissues in the TCGA cohort. M0 macrophages had obviously negative correlation with CD4 memory resting T cells (Cor = −0.63), Tfh cells (Cor = −0.64) and M1 macrophages (Cor = −0.61). Interestingly, Tfh cells and M1 macrophages were most relevant in CRC samples (Cor = 0.52).

Moreover, the BTLA’s role in regulating tumor immune cells was qualified by the correlation between BTLA expression and the immune cells gene markers in CRC. First, gene markers of general T cells, like CD3E, CD3G, CD28, and CD2, showed strong correlations with BTLA expression. In addition, CD8A in CD8T cells was also strongly related to BTLA expression (Table 3), confirming the theory that the BTLA-HVEM complex signaling system promotes the tumor antigen-specific CD8 + T cells’ survival (Derre et al., 2010). IL-21, a crucial protein to identify Tfh cells, was highly correlated with BTLA expression in CRC in our analysis. Correlation result between BTLA and markers of Tfh cells was similar to the research that BTLA inhibits the secretion of IL-21 by Tfh to inhibit IgG production (Kashiwakuma et al., 2010). Previous researches of BTLA’s function has been focused on TCR or T cells mostly, but few studies on B cells’ role. Our results indicated that gene markers of B cells like CD19 and CD79A were strongly correlated with BTLA expression (Table 3). Studies also suggested that BTLA is a kind of inhibitory receptor in the B cell receptor (BCR) signaling pathway (Vendel et al., 2009; Thibult et al., 2013). Most gene makers of NK cells showed mild and moderate correlations with BTLA expression in our research, supporting that BTLA limited anti-tumor immunity through type I NKT cells (Sekar et al., 2018). These results revealed the potential effect of BTLA in B cells, T cells, and NK cells to maintain the homeostasis of the colorectal TME.

**TABLE 3:** Correlation analysis between BTLA and relate genes and markers of immune cells in CRC.

Description	Gene markers	Pearson *r*	*p*	Number of Pairs
B cell	CD19	0.82	****	610
	CD79A	0.76	****	610
T cell (general)	CD3E	0.78	****	610
	CD3G	0.68	****	610
	CD28	0.81	****	610
	CD2	0.63	****	610
CD8 + T cell	CD8A	0.52	****	610
	CD8B	0.28	****	610
Helper T cell	CD4	0.56	****	610
	CD40LG (CD40L)	0.65	****	610
	CXCR4	0.49	****	610
Th1	T-bet (TBX21)	0.56	****	610
	STAT1	0.29	****	610
	STAT4	0.68	****	610
	IFN-γ (IFNG)	0.29	****	610
	TNF-α (TNF)	0.22	****	610
Th2	GATA3	0.47	****	610
	STAT6	0.00	ns	610
	STAT5A	0.33	****	610
	IL13	0.13	*	379
Th17	STAT3	0.26	****	610
	IL17A	−0.02	ns	379
Tfh	BCL6	0.35	****	610
	IL21	0.65	****	379
Treg	FOXP3	0.41	****	610
	CCR8	0.39	****	610
	STAT5B	0.22	****	610
	TGFβ (TGFB1)	0.28	****	610
	IL2RA (CD25)	0.47	****	610
Natural killer cell	FCGR3A (CD16a)	0.35	****	610
	NCAM1 (CD56)	0.30	****	610
	KIR2DL1	0.14	**	379
	KIR2DL3	0.15	***	610
	KIR2DL4	0.26	****	610
	KIR3DL1	0.12	**	610
	KIR3DL2	0.21	****	610
	KIR3DL3	0.07	ns	379
	KIR2DS4	0.15	***	610
Monocyte	CD86 (B7-2)	0.39	****	610
	CD115 (CSF1R)	0.40	****	610
TAM	CCL2	0.29	****	610
	CD68	0.23	****	610
	IL10	0.33	****	610
M1 Macrophage	NOS2	0.02	ns	610
	IRF5	0.10	*	610
	PTGS2	0.01	ns	610
M2 Macrophage	CD163	0.33	****	610
	VSIG4	0.32	****	610
	MS4A4A	0.41	****	610
Dendritic cell	HLA-DPA1	0.42	****	610
	HLA-DPB1	0.47	****	610
	HLA-DQB1	0.34	****	610
	HLA-DRA	0.38	****	610
	BDCA-1 (CD1C)	0.58	****	610
	BDCA-4 (NRP1)	0.36	****	610
	CD11c (ITGAX)	0.36	****	610
Neutrophils	CD66b (CEACAM8)	−0.05	ns	610
	CD11b (ITGAM)	0.37	****	610
	CCR7	0.85	****	610

CRC, colorectal cancer; Th, T helper cell; Tfh, Follicular helper T cell; Treg, regulatory T cell; TAM, tumor-associated macrophage; ns, p > 0.05; *p < 0.05; **p < 0.01; ***p < 0.001; ****p < 0.0001.

### Functional Analysis and Predicted Signaling Pathways of BTLA

**FIGURE 4:**
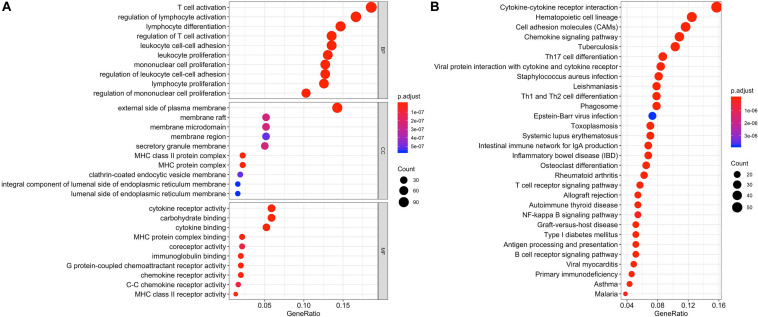
Function enrichment analysis of the co-expressed genes of BTLA. KEGG and GO biological process enrichment of BTLA co-expressed genes **(A)** The top 10 enrichment results of the BP, CC, and MF category. **(B)** The top 30 enrichment results of the KEGG signal pathway. Clusters are based on the gene count to measure the similarity between terms. BP, biological process; CC, cellular component; MF, molecular function; KEGG, Kyoto Encyclopedia of Genes and Genomes.

Differentially expressed genes and co-regulated in a biological state, are more likely to uncover the BTLA’s specific mechanisms. As illustrated in Figures 4A,B, functional enrichment analyses were performed. The top 10 enrichment results (ranged by count) in biological process (BP), cellular component (CC), and molecular function (MF) were further clustered and established by bubble plots (Figure 4A). The results visualized that BTLA was integral to the external side of the plasma membrane and intrinsic to the membrane and the plasma membrane part. In addition, BTLA markedly participated in the regulation of leukocyte cell adhesion, activation, and proliferation. We affirmed that biological processes T cell activation and regulation of T cell activation were most likely mechanisms to represent the BP of BTLA in CRC. Similarly, BTLA involved in B cell differentiation, activation, regulation (Supplementary Table S1).

In the top 30 KEGG pathway terms, most of the involved significant pathways included cytokine-cytokine receptor interaction, hematopoietic cell lineage, cell adhesion molecules, chemokine signaling pathway. Besides, Th1, Th2, and Th17 cell differentiation were also enriched terms (ranged by count; Figure 4B) in the KEGG results, and details were shown in Supplementary Table S2.

Therefore, combining previous studies and the above-mentioned results, we concluded that the TCR signaling pathway, BCR signaling pathway, and NK cell-mediated cytotoxicity might be indicative of a potential mechanism in which BTLA was involved in CRC.

## Discussion

BTLA, also named CD272, was first discovered by inhibiting Th1 cell expression in 2003. It is the thirdly discovered molecule of the CD28 family behind PD-1 and CTLA-4 (Watanabe et al., 2003). Studies established the singular role of BTLA as both co-stimulator and co-inhibitor to activated cancerous CD8 + T cells (Derre et al., 2010; Ritthipichai et al., 2017). The clinical trial in which the registration number was NCT00854399 revealed that BTLA detected in epithelial ovarian carcinoma (EOC) tissues can predict poor outcomes of patients, and chemotherapy combined with BTLA inhibitor can enhance immune activation and produces effective anti-tumor effects (Chen Y. L. et al., 2019). So far, it remains inconclusive about the mechanism of BTLA in CRC.

In our study, we analyzed the BTLA expression samples among TIMER, Oncomine, and TCGA databases, suggesting that BTLA was lower in CRC tissues compared to the normal tissues. In the TCGA and GEO cohorts, the upregulation of BTLA expression correlated with a good prognosis. Then we analyzed the clinical information in TCGA datasets to explore the detailed mechanisms and potential relationship of BTLA expression in CRC. The Chi-Squared test revealed that BTLA expression was correlated to lymph node metastasis. In our multivariable Cox regression model, BTLA expression was an independent prognostic factor in patients with CRC.

Our study concluded a critical conclusion that the BTLA expression was significantly associated with immune infiltration levels in CRC. We used CIBERSORT R package for statistical analysis, indicating a significant association between BTLA expression and immune infiltration levels of B cells, T cells, mast cells, monocytes, NK cells, and macrophages in CRC. Correlation analysis of immune cell gene markers and BTLA expression confirmed previous studies. These results indicated that BTLA might regulate B cells, T cells, and NK cells in the colorectal tumor immune microenvironment.

To investigate the mechanisms of BTLA immune regulation in CRC, we used BTLA co-expressed genes for GO and KEGG enrichment analysis. Thus, we concluded that the TCR signaling pathway, BCR signaling pathway, and NK cell-mediated cytotoxicity pathway were the particular mechanism for BTLA mediating the immune infiltration of CD8 T cells, Tfh cells, B cells and NK cells in CRC. Together these functional analyses suggested that the BTLA plays a vital role in the balance and regulation of TIICs in CRC.

Immunotherapy is an auspicious and practical treatment for some patients. Prevenient studies have demonstrated that the level of tumor infiltration by CD8 + T cells can predict patient clinical prognosis in melanoma, ovarian, and CRC (Giraldo et al., 2019). In this article, higher BTLA expression was positively associated with the CD8 cells’ immune infiltration level and better outcomes. However, some research concluded that CRC might not be suitable for immunotherapy (Banerjea et al., 2004; Boland and Ma, 2017). We revealed that BTLA could be a positive stimulatory factor or an indispensable regulator in the CRC microenvironment. Based on the results of this article, we inferred BTLA could be a potential target of CRC immunotherapy in developing novel immunological treatment strategies for CRC.

There are certain limitations to our research. First, the number of samples being screened for cox regression analysis is too small, only transcriptomics expression of BTLA with full clinical data was analyzed in this study. Second, cytokines not only carry information between tumors and immune cells but also be secreted by immune cells, playing an important role in TME (Burkholder et al., 2014; Zhang J. et al., 2019). Cytokines-cytokines receptor interaction pathway is the top terms in KEGG analysis, but the role of BTLA in this pathway needs further validation. Third, underlying mechanisms of immune infiltration signaling pathways in CRC remain unclear, while function annotations and enrichment analysis of BTLA are investigated. Future research is required to explore the elaborate mechanism of BTLA in CRC and investigate the functions of BTLA in other cancers.

In summary, higher BTLA expression correlated with extended outcome and increased immune infiltration levels in naive B cells, memory B cells, CD8 T cells, CD4 memory resting T cells, Tfh cells, resting NK cells, M1 macrophages and resting mast cells in CRC microenvironment. In addition, BTLA expression potentially contributes to the regulation of B cells, NK cells, T cells, especially CD8 T cells and Tfh through TCR signaling pathway, BCR signaling pathway, and NK cell-mediated cytotoxicity pathway. Therefore, BTLA plays an essential role in immune cell infiltration and functions as a prognosis biomarker and may be helpful in developing novel immunological treatment strategies for CRC.

## Data Availability Statement

Publicly available datasets were analyzed in this study, these can be found in The Cancer Genome Atlas (https://portal.gdc.cancer.gov/) and the NCBI Gene Expression Omnibus (GSE29623 and GSE17536).

## Author Contributions

Both authors listed have made a substantial, direct and intellectual contribution to the work, and approved it for publication.

## Conflict of Interest

The authors declare that the research was conducted in the absence of any commercial or financial relationships that could be construed as a potential conflict of interest.

## Supplementary Material

FIGURE S1 The multivariate Cox analysis of BTLA expression and clinicopathological factors.

TABLE S1 Gene ontology of BTLA.

TABLE S2 Kyoto Encyclopedia of Genes and Genomes of BTLA.

## Abbreviations

AJCC: the American Joint Committee on Cancer
BCR: B cell receptor
BLCA, bladder cancer;BTLA: B and T lymphocyte attenuator
CIBERSORT: Cell-type identification by estimating relative subsets of RNA transcripts
COAD: colon adenocarcinoma
CRC: colorectal cancer
CTLA-4: Cytotoxic T lymphocyte antigen 4
DFI: disease-free interval
DSS: disease-specific survival
EOC: epithelial ovarian carcinoma
GEO: Gene Expression Omnibus
GO: gene ontology
HVEM: Herpes virus entry mediator
ICOS: inducible costimulatory molecule
KEGG: Kyoto Encyclopedia of Genes and Genomes
KIRC: kidney renal clear cell carcinoma
KIRP: kidney renal papillary cell carcinoma
LUAD: lung adenocarcinoma
LUSC: lung squamous cell carcinoma
MMR: mismatch repair
MSI-H: high microsatellite instability
NK cell: natural killer cells
OS: overall survival
PD-1: programmed death-1
READ: rectum adenocarcinoma
TIICs: tumor infiltrating immune cells
TME: tumor microenvironment
TIMER: Tumor Immune Estimation Resource
TCR: T-cell receptor
TCGA: the cancer genome atlas
THCA: thyroid carcinoma
Tfh cell: follicular helper T
Th cell: T helper cells
PFI: progression-free interval
TNM staging system: the tumor, node, metastasis staging system.
